# Beyond body mass index: the role of fat distribution in male sperm quality

**DOI:** 10.3389/fendo.2025.1702791

**Published:** 2025-10-23

**Authors:** Dongsheng Ma, Mengru Zhang, Xiaoguang Zhang, Lizhen Xu

**Affiliations:** ^1^ Department of Reproductive Medicine, The Affiliated Bozhou Hospital of Anhui Medical University, Bozhou, China; ^2^ Department of Reproductive Medicine, The People’s Hospital Bozhou, Bozhou, China; ^3^ Andrology and Embryology Laboratory, The Affiliated Bozhou Hospital of Anhui Medical University, Bozhou, China; ^4^ Andrology and Embryology Laboratory, The People’s Hospital Bozhou, Bozhou, China; ^5^ Department of Burns and Plastic Surgery, Ya’an People’s Hospital, Ya’an, China

**Keywords:** sperm dynamics parameters, sperm morphology parameters, obesity, central obesity, male fertility

## Abstract

**Objective:**

To explore the dual role of obesity and fat distribution on sperm dynamics and morphological parameters and to further assess the impact on male fertility.

**Methods:**

A population of 823 male semen examinations from the Male Reproductive Health Database (FAST-Date, 2022-2025), was retrospectively analyzed for general information, obesity indicators, sperm dynamics and morphology parameter ratings, and male fertility assessment indicators.

**Results:**

There were differences in sperm dynamics and sperm morphology parameters between the non-obesity and obesity group populations (*P* < 0.05), which were shown to be poorer in both sperm dynamics parameters in the obesity group population as compared to the non-obesity group population, and morphological parameters. There were differences in total sperm count, sperm concentration, sperm dynamics parameters and sperm morphology parameters among obesity subgroups, and central obesity showed that sperm dynamics and morphology parameters were better than those of generalized obesity and simple obesity groups. And obesity group had higher sperm DFI compared to non-obesity group (23.83 ± 12.25 vs. 14.16 ± 9.80), whereas there was no statistically significant difference in sperm DFI between obesity subgroups (*P* = 0.210). Multivariate regression analysis showed that PR was significantly negatively associated with the risk of male infertility (adjusted OR, 0.93; 95% CI, 0.89-0.98; *P* = 0.004). hyperactivated spermatozoa revealed significant associations with the adverse pregnancy outcomes (adjusted OR, 0.93; 95% CI, 0.87–1.00; *P* = 0.049). A significant direct effect of obesity on sperm DFI was observed (β= -9.67, 95% CI: -11.19~-8.15, *P* < 0.001), while DFI itself was a significant predictor of adverse pregnancy outcomes (β=-0.02, 95% CI: -0.04~-0.01, *P* = 0.029).

**Conclusion:**

Obesity reduces sperm quality (sperm dynamics and morphological parameters), whereas central obesity outperforms generalized and simple obesity in some sperm dynamics and morphological parameters. This underscores the clinical importance of assessing fat distribution, not just overall obesity, in the evaluation of male reproductive health.

## 1 Introduction

Between 1990 and 2021, overweight and obesity prevalence increased in all countries and regions globally, with an estimated 100 million adult males overweight and obese by 2021 (1). The overweight and obesity situation facing China is equally bleak, with an overall prevalence of obesity of 8.1%, of which an estimated 48 million adult males are obese in 2018 (2). The age-standardized prevalence rates of central obesity only, generalized obesity only, and both central and generalized obesity among Chinese adults aged 18–65 years have increased, respectively, from 15.8% in 1993, 0.2% and 2.9% to 30.3%, 0.9% and 10.3% in 2011 (3). The age-standardized prevalence of central obesity in Chinese adults with BMI <25 kg/m² ranged from 21.1% to 30.3% (3–5). Infertility is a condition in which clinical pregnancy outcome is not achieved at 12 months without the use of contraception. It is estimated that infertility affects between 8% and 12% of couples of reproductive age globally, with male infertility contributing to approximately 20-30% (6). Male sperm concentration (SC) has shown a significant downward trend globally (7, 8). In Chinese men, SC and total sperm count (TSC) also show significant decreasing characteristics, suggesting the existence of serious reproductive warnings in men (9). TSC decreases with age. Although this decline may be due to a multifactorial cumulative effect that has not yet been fully elucidated, potential causes include increased rates of obesity, and unhealthy eating patterns (10). Central obesity, also known as abdominal obesity and visceral obesity, is a type of obesity in which fat accumulates predominantly in the abdomen and around the internal organs. The existing evidence regarding the association between obesity and semen parameters is inconsistent. While some studies demonstrated that SC, TSC, total viability, and normal morphology are lower in obese men compared to normal weight healthy subjects (11), others refute these findings (12, 13). This discrepancy underscores the need for further investigation into the potential relationship between adiposity and male reproductive function. It was found that sperm parameters (SC, TSC, and semen volume) were differentially decreased in overweight or obesity as compared to normal weight and there was a relationship between BMI and sperm quality suggesting that obesity may be a deleterious factor in male infertility (14). Obesity has a negative impact on various parameters of male fertility, ranging from semen quality to sperm DNA integrity, and male obesity is negatively associated with live birth rates in pregnancies conceived naturally and with assisted reproductive technologies (15). In this study, we investigated the effects of obesity on sperm motility and sperm morphology.

## 2 Methods

### 2.1 Study subjects

Data from 11,983 men in the male reproductive health database of Bozhou, Anhui Province, China (The People’s Hospital Bozhou, FAST-Date, 2022-2025), a total of 823 men were included in the study by inclusion and exclusion criteria, and were retrospectively analyzed for general information, obesity-related indices, sperm dynamics and morphological parameters, sperm DNA fragmentation indices(DFI), and indicators of fertility assessment.

### 2.2 Inclusion and exclusion criteria

This study applied uniform inclusion and exclusion criteria to screen eligible participants. All participants were required to meet the following general inclusion criteria: (1) male, aged between 18 and 50 years; (2) agreement to participate in the study and provision of signed informed consent, along with an information letter detailing the potential benefits and risks; and (3) availability of complete clinical and demographic data.

To minimize potential bias and confounding factors, individuals meeting any of the following conditions were excluded: (1) chromosomal abnormalities, genetic or familial disorders; (2) diagnosis of azoospermia, or cryptozoospermia; (3) missing key data such as demographic or clinical information; or (4) presence of ovulatory disorders, reproductive tract abnormalities, or genetic issues in the spouse.

Furthermore, known confounders that could potentially influence the study outcomes were also grounds for exclusion. These included: (1) metabolic disorders such as diabetes mellitus, hypertension, and moderate-to-severe hyperlipidemia; (2) reproductive system disorders, including moderate-to-severe varicocele (confirmed clinically or by ultrasonography), orchitis, and cryptorchidism; (3) adverse lifestyle and behavioral factors, namely alcoholism and tobacco addiction; (4) medication effects, such as the use of endocrine-modulating drugs or exogenous testosterone supplementation; and (5) Male hypogonadism, high FSH or low testosterone blood.

### 2.3 Study data

#### 2.3.1 General information

Age, hypertension, diabetes mellitus, sex hormone (T, E2, FSH, PRL, LH), testicular volume, body mass index (BMI), waist circumference (WC).

#### 2.3.2 Sperm quality assessment indicators

Diagnostic criteria are based on the World Health Organization (WHO) (WHO Laboratory Manual for the Examination and Processing of Human Semen, 5th edition).

1. Sperm routine parameters: semen volume, total sperm count, sperm concentration.
2. Sperm dynamics parameters: sperm dynamics are assessed using the computer aided semen analysis system (CASA). Classification of motility patterns: progressive motility (PR%), hyperactivated spermatozoa. Motion velocity indicators: curve velocity (VCL) μm/s, straight line velocity (VSL) μm/s, average path velocity (VAP) μm/s. Characteristic parameters of motility: Straightness (STR), beat-cross frequency (BCF).
3. Sperm morphology parameters: Sperm morphology was assessed using the WHO recommended Diff-Quik method. Overall sperm morphology parameters: Normal sperm morphology rate (%). Morphological defect parameters: Head abnormalities (HAB), midpiece abnormalities (MAB), principal piece abnormalities (PAB), and cytoplasmic abnormalities (CAB). Morphological characterization parameters:Teratozoospermia index (TZI), sperm deformity index (SDI).
4. Sperm DNA fragmentation index (DFI): Sperm DFI was assessed using sperm chromatin structure assay (SCSA).

#### 2.3.3 Fertility assessment indicators

Spousal adverse pregnancy outcomes (APO): unexplained spontaneous abortions, biochemical pregnancies, embryonic sterilization, etc.; Male infertility (MI): failure to use contraception, regular sexual intercourse, and failure to achieve a clinical pregnancy outcome at 12 months.

### 2.4 Study definitions

Obesity was defined as BMI ≥ 28 kg/m^2^ or WC for men ≥ 90 cm, from The Working Group on Obesity in China (WGOC). Oligozoospermia: sperm concentration < 15x10^6^/mL or total sperm count< 39x10^6^;. Asthenospermia definition: PR < 32%. Teratospermia definition: normal sperm morphology rate: < 4%.

### 2.5 Study protocol

The included subjects were divided into obese group (obesity group, N = 310) and non-obesity group (non-obesity group,N=513) according to the study definition. Subgroup analyses of the obesity group were performed according to the subgroup criteria: generalized obesity group (N = 233), simple obesity group (N = 17), and central obesity group (N = 60); sperm routine parameters, sperm dynamics parameters, sperm morphology parameters and sperm DFI were analyzed.

Obesity was classified into three types: central obesity (BMI < 28 kg/m^2^ and WC ≥ 90cm), simple obesity (BMI ≥ 28 kg/m^2^ and WC < 90cm), and generalized obesity (BMI ≥ 28 kg/m^2^ and WC ≥ 90cm). BMI < 28 kg/m^2^ and WC < 90 cm were considered normal weight, non-obesity.

### 2.6 Quality control

1. Data quality control: Standardized data collection and management system (FAST-Date), regional network-based Electronic Data Capture (EDC), and in-process QC and on-line QC for data collection and management.
2. Sample quality control: CASA (China, Beijing, Suijia software SSA-II) was used for semen analysis, with the frame rate of video acquisition set at 60Hz and at least 200 sperm tracks per sample. More than 200 sperm/samples were independently evaluated by two male laboratory experts who passed the External Quality Assessment (EQA, China Association for Maternal and Child Health), and a third expert arbitrated any disagreement. Sperm dynamics parameters were archived with instantaneous images and sperm morphology was scored with a multi-parameter morphology score combined with a weighted scoring system for defect type and severity.
3. Quality control of testing methods: semen testing strictly follows international and domestic standards: ① WHO Manual for Human Semen Examination (5th edition); ② ISO 23162:2021 specification of CASA system performance verification methods; ③ China’s quality control standards for semen analysis ④ The laboratory has passed ISO 15189 certification and the certification of the training base of human sperm bank of China National Health and Wellness Commission, which ensures the traceability of test results and the consistency of indoor and inter-laboratory.

### 2.7 Ethics review

This retrospective study has been received ethical approval (No. BY-2025-134). All participants signed informed consent forms.

### 2.8 Statistical methods

SPSS23.0 statistical software was used for data processing and analysis. Measurement information was tested by t-test between groups, and ANOVA was used for comparison between multiple groups; and χ2 test was used for comparison between groups; measurement information conforming to normal distribution was expressed by mean ± standard deviation, and t-test of independent samples was used for comparison between groups; measurement information not conforming to normal distribution was expressed by M (P_25_, P_75_), and non-parametric Mann-Whitney U test; and comparisons between groups were made using the X² test; Univariate analysis and multivariate analysis: multivariate logistic regression analysis was performed to determine the independent factors significantly associated with adverse pregnancy outcomes and male infertility, while adjusting for potential confounders. All variables from the univariate analysis were included in the multivariate Logistic regression model. Test level α = 0.05.

### 2.9 Study flow chart

**Figure 1:**
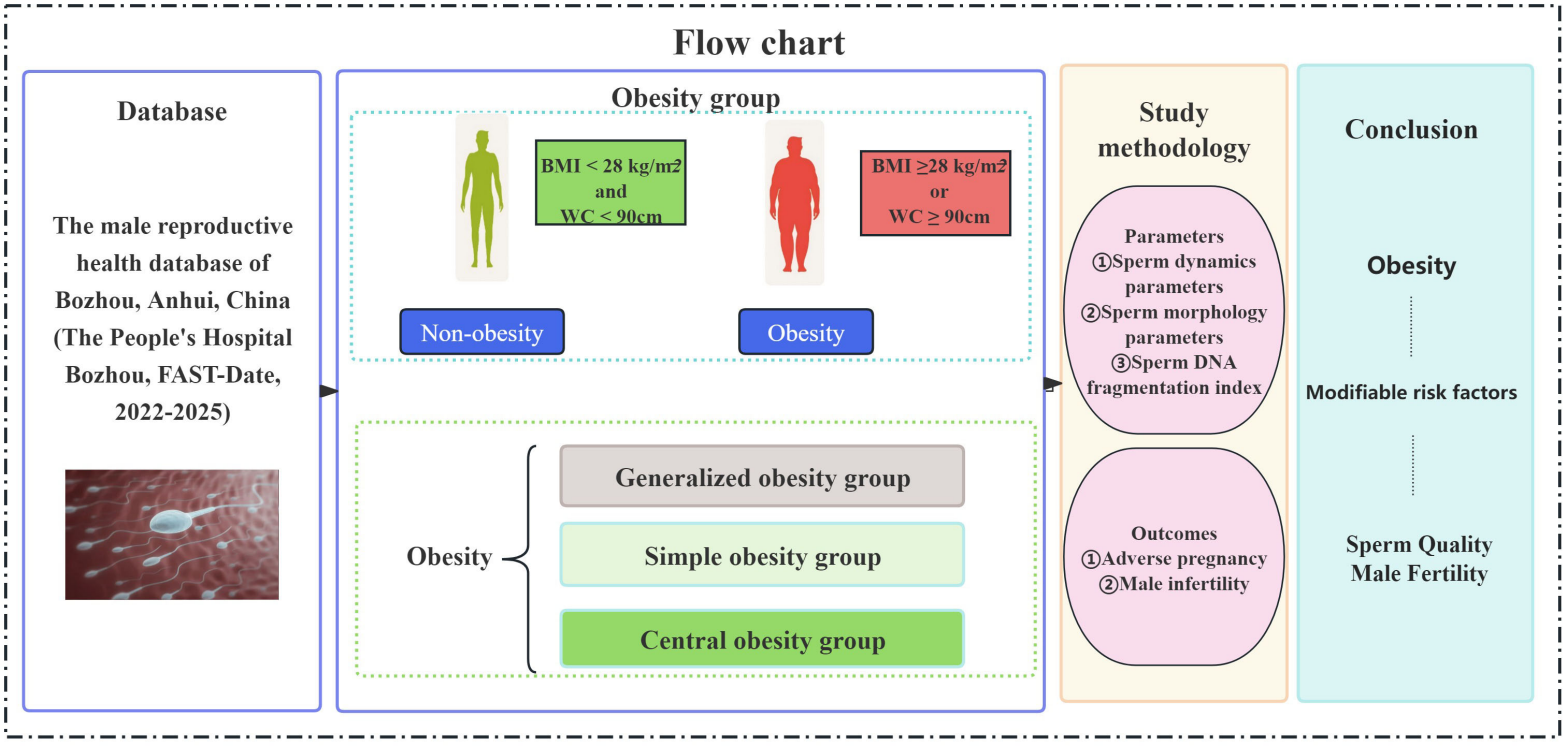
Flow chart.

A total of 823 male subjects were included in the study. The flow chart of the study selection process is shown in **Figure 1**. The subjects were divided into non-obesity and obesity groups according to BMI and WC. The obesity group was further divided into three subtypes: generalized obesity, simple obesity and central obesity. The correlations between each group and sperm dynamics and morphology parameters, and sperm DFI were analyzed and compared.

## 3 Results

### 3.1 Comparison of sperm dynamics and morphological parameters in obesity and obesity subgroups

Overall age of this study population was 30.23 ± 5.03 years (N = 823), age of non-obesity group was 29.07 ± 4.61 years (N = 513) and age of obesity group was 32.16 ± 5.11 years (N = 310) (*P* < 0.001). The age of generalized obesity group was 32.02 ± 4.97 years (N = 233), age of simple obesity group was 32.59 ± 5.86 years (N = 17), age of central obesity group was 32.58 ± 5.49 years (N = 60).There was no statistically significant difference between the two comparisons(*P*>0.05).

There was no statistically significant difference in total sperm count and sperm concentration between the non-obesity and obesity group populations, while there were differences in sperm dynamics parameters (PR, VCL, VSL, VAP, STR, BCF) and sperm morphology parameters (normal sperm morphology rate, HAB, MAB, PAB, TZI, SDI) (*P* < 0.05), which were shown to be poorer in both sperm dynamics parameters in the obesity group population as compared to the non-obesity group population, and morphological parameters. See **Table 1**.There were differences in total sperm count, sperm concentration, sperm dynamics parameters (PR, VCL, VSL, VAP, STR, BCF) and sperm morphology parameters (normal sperm morphology rate) among obesity subgroups, where central obesity group differed from generalized obesity and simple obesity groups in total sperm count, sperm concentration and normal sperm morphology rate(*P* < 0.05), which were more superior, see **Table 2**.

**Table 1:** Comparison of sperm dynamics and morphology parameters between obesity group and non-obesity group.

Groups	Sperm dynamics parameters							
	Total sperm count	Sperm concentration	PR%	VCL	VSL	VAP	STR	BCF
Obesity group	316.13(171.01,565.95)	89.09(46.66,143.86)	45.81 (29.01,59.81)	27.42 ± 14.87	12.67 ± 7.10	18.11 ± 9.42	0.37 ± 0.15	7.40 ± 2.83
Non-obesity group	331.18(181.55,551.58)	95.09(53.10,159.30)	52.11 (39.13,65.54)	32.29 ± 15.06	15.02 ± 7.38	21.31 ± 9.65	0.42 ± 0.15	8.33 ± 2.96
t/Z	-0.230	1.715	4.569	4.515	4.484	4.653	4.218	4.452
*P*	0.818	0.086	<0.001	<0.001	<0.001	<0.001	<0.001	<0.001

Sperm morphology parameters								
Groups	Normal sperm morphology rate %	HAB	MAB	PAB	CAB	TZI	SDI	
Obesity group	3.29 ± 1.60	92.10 ± 4.31	29.52 ± 6.17	20.54 ± 5.86	2.93 (0.49,5.76)	1.51 ± 0.12	1.46 ± 0.13	
Non-obesity group	3.77 ± 2.08	91.15 ± 4.61	27.64 ± 6.95	18.73 ± 5.79	2.93 (0.95,5.57)	1.46 ± 0.13	1.41 ± 0.14	
t/Z	3.688	-2.944	-4.028	-4.352	-0.029	-4.710	-4.660	
*P*	<0.001	0.003	<0.001	<0.001	0.977	<0.001	<0.001	

PR, Progressive Motility; VCL, Curve Velocity; VSL, Straight Line Velocity; VAP, Average Path Velocity; STR, Straightness; BCF, Beat-CrossFrequency; VSL, Straight Line Velocity.

HAB, Head Abnormalities; MAB, Midpiece Abnormalities; PAB, Principal Piece Abnormalities; CAB, Cytoplasmic Droplets; TZI, Teratozoospermia Index; SDI, Sperm Deformity Index.

**Table 2:** Comparison of sperm dynamics and morphology parameters between obesity subgroups.

Subgroups	Sperm dynamics parameters							
	Total sperm count	Sperm concentration	PR%	VCL	VSL	VAP	STR	BCF
Generalized obesity group	270.18 (145.57,496.13)	74.06(41.24,121.53)	44.05 (28.50,57.97)	25.94 ± 14.38	11.97 ± 6.75	17.19 ± 9.06	0.36 ± 0.14	7.11 ± 2.80
Simple obesity group	228.10(145.19,355.48)	61.54(39.11,75.39)	45.83 (26.92,57.19)	26.59 ± 13.22	12.34 ± 6.62	17.74 ± 8.48	0.36 ± 0.13	7.37 ± 2.57
Central obesity group	579.40 (354.41,973.87)^C^	150.86 (118.31,206.18)^C^	53.26 (42.49,66.33)	33.42 ± 15.85	15.47 ± 7.96	21.79 ± 10.26	0.43 ± 0.14	8.55 ± 2.79
*F/H*	33.395	49.882	8.618	6.264	6.006	5.884	6.644	6.389
*P*	<0.001	<0.001	0.013	0.002	0.003	0.003	0.001	0.002

Sperm morphology parameters								
Subgroups	Normal sperm morphology rate %	HAB	MAB	PAB	CAB	TZI	SDI	
Generalized obesity group	3.20 ± 1.45	92.28 ± 4.10	29.87 ± 6.02	20.73 ± 5.65	2.93 (0.49,5.84)	1.51 ± 0.11	1.46 ± 0.12	
Simple obesity group	2.89 ± 1.36	92.70 ± 5.17	30.26 ± 5.68	21.73 ± 6.42	1.99 (0,7.00)	1.53 ± 0.14	1.49 ± 0.15	
Central obesity group	3.77 ± 2.09^C^	91.24 ± 4.82	27.95 ± 6.69	19.52 ± 6.41	3.06 (0.61,5.56)	1.48 ± 0.14	1.42 ± 0.16	
*F/H*	3.761	1.571	2.471	1.389	0.151	2.222	2.677	
*P*	0.024	0.209	0.086	0.251	0.927	0.110	0.070	

Two-by-two comparisons were made between the three groups and corrected for *P*-values.

a Statistically significant difference between the generalized obesity group and the other subgroups; Statistically significant difference between the simple obesity group and the other subgroups; Statistically significant difference between the central obesity group and the other subgroups.

### 3.2 Comparison of sperm DNA fragmentation index in obesity and obesity subgroups

There was a statistically significant difference in sperm DFI between obesity and non-obesity groups (*P* < 0.001), and obesity group had higher sperm DFI compared to non-obesity group (23.83 ± 12.25 vs. 14.16 ± 9.80), whereas there was no statistically significant difference in sperm DFI between obesity subgroups (*P* = 0.210), where two comparisons between the three groups were also not statistically different (*P*>0.05), see **Table 3**.

**Table 3:** Comparison of sperm DFI between subgroups with obesity.

Characteristic	Sperm DFI	*t/F*	*P*
Groups		-12.46	<0.001***
Non-obesity group	14.16 ± 9.80		
Obesity group	23.83 ± 12.25		
Subgroups			
Generalized obesity group	24.44 ± 12.23	1.571	0.210
Simple obesity group	24.35 ± 10.56		
Central obesity group	21.31 ± 12.64		

*P < 0.05; **P < 0.01; ***P < 0.001.

### 3.3 Comparison of clinical symptom stratification in obesity and obesity subgroups

No statistically significant differences were observed between the obesity and non-obesity groups regarding the presence of male infertility (57.7% vs. 56.3%, *P* = 0.693) or adverse pregnancy outcomes in partners (22.6% vs. 20.3%, *P* = 0.432). Similarly, the prevalence of oligozoospermia was comparable between groups (1.6% vs. 1.9%, *P* = 0.727). However, significant differences were noted for asthenospermia and teratospermia: the obesity group demonstrated a higher proportion of asthenospermia (26.1% vs. 14.2%, *P* < 0.001) and teratospermia (70.3% vs. 61.4%, *P* = 0.009) compared to the non-obesity group. See **Table 4**.

**Table 4:** Comparison of clinical symptom stratification between obesity and non-obesity groups.

Characteristic	Obesity group N = 310	Non-obesity group N = 513	χ²	*P*
Male infertility, n (%)			0.16	0.693
No	131 (42.3%)	224 (43.7%)		
Yes	179 (57.7%)	289 (56.3%)		
Adverse pregnancy outcomes, n (%)			0.62	0.432
No	240 (77.4%)	409 (79.7%)		
Yes	70 (22.6%)	104 (20.3%)		
Oligozoospermia, n (%)			0.12	0.727
No	305 (98.4%)	503 (98.1%)		
Yes	5 (1.6%)	10 (1.9%)		
Asthenospermia, n (%)			17.99	<0.001
No	229 (73.9%)	440 (85.8%)		
Yes	81 (26.1%)	73 (14.2%)		
Teratospermia, n (%)			6.74	0.009
No	92 (29.7%)	198 (38.6%)		
Yes	218 (70.3%)	315 (61.4%)		

Clinical symptom stratification of the study participants stratified by obesity subgroups are presented in **Table 5**. The prevalence of male infertility with no statistically significant difference observed across subgroups (*P* = 0.555). Similarly, the proportion of participants with adverse pregnancy outcomes in their partners did not differ significantly among the three subgroups (*P* = 0.878). Oligozoospermia was rare across all subgroups (*P* = 0.292). Asthenospermia the differences were not statistically significant (*P* = 0.290). Teratospermia showing a trend toward higher prevalence in the central obesity group, although this did not reach statistical significance (*P* = 0.074), However, the central obesity group showed a statistically significant difference in teratospermia compared to the other two subgroups.

**Table 5:** Comparison of clinical symptom stratification between obesity subgroups.

Characteristics	Central obesity group N = 60	Simple obesity group N = 17	Generalized obesity group N = 233	H	*P*
Male infertility, n (%)				1.18	0.555
No	23 (38.3%)	9 (52.9%)	99 (42.5%)		
Yes	37 (61.7%)	8 (47.1%)	134 (57.5%)		
Adverse pregnancy outcomes, n (%)				0.28	0.878
No	48 (80.0%)	13 (76.5%)	179 (76.8%)		
Yes	12 (20.0%)	4 (23.5%)	54 (23.2%)		
Oligozoospermia, n (%)				2.10	0.292
No	59 (98.3%)	16 (94.1%)	230 (98.7%)		
Yes	1 (1.7%)	1 (5.9%)	3 (1.3%)		
Asthenospermia, n (%)				2.35	0.290
No	49 (81.7%)	12 (70.6%)	168 (72.1%)		
Yes	11 (18.3%)	5 (29.4%)	65 (27.9%)		
Teratospermia, n (%)				5.20	0.074
No	25 (41.7%)	4 (23.5%)	63 (27.0%)		
Yes	35 (58.3%)	13 (76.5%)	170 (73.0%)		

### 3.4 Multivariate regression analysis of male infertility and adverse pregnancy outcomes

Results of multivariate regression analysis showed that sperm PR was significantly negatively associated with the risk of male infertility (adjusted OR, 0.93; 95% CI, 0.89-0.98; *P* = 0.004). Other sperm dynamics and morphology parameters, including DFI (adjusted OR, 0.99; 95% CI. 0.97-1.00; *P* = 0.052), and multiple sperm morphology parameters (HAB, MAB, PAB, CAB) did not show statistically significant associations (all *P* > 0.05). Obesity-related indices (obesity and obesity classification) also did not show significant associations with male infertility. See **Table 6**.

**Table 6:** Univariate and multivariate analysis of influencing factors (Logistic regression).

Characteristics	Univariable			Multivariable		
	OR	95% CI	*P*	OR	95% CI	*P*
Groups of Obesity						
Non-obesity group	0.94	0.71, 1.26	0.693			
Obesity group	1.00	0.74, 1.34	0.980			
Subgroups of Obesity						
Central obesity group	1.25	0.72, 2.16	0.431			
Simple obesity group	0.69	0.26, 1.81	0.451			
Generalized obesity group	1.05	0.77, 1.43	0.764			
Oligozoospermia	0.50	0.18, 1.42	0.192	0.68	0.23, 2.03	0.490
Asthenospermia	0.76	0.53, 1.08	0.123	0.68	0.41, 1.13	0.138
Teratospermia	0.69	0.51, 0.92	0.012*	1.02	0.68, 1.54	0.923
DFI	0.99	0.97, 1.00	0.017*	0.99	0.97, 1.00	0.052
Semen volume	1.00	1.00, 1.00	0.597	1.00	1.00, 1.00	0.587
Total sperm count	0.98	0.91, 1.05	0.570	1.00	0.88, 1.14	0.969
Sperm concentration	1.00	1.00, 1.00	0.270	1.00	1.00, 1.00	0.638
PR	1.00	1.00, 1.01	0.320	0.93	0.89, 0.98	0.004**
Hyperactivated spermatozoa	1.02	1.00, 1.04	0.042*	1.00	0.94, 1.06	0.960
VCL	1.01	1.00, 1.02	0.157	1.01	0.81, 1.24	0.957
VSL	1.02	1.00, 1.04	0.065	0.95	0.65, 1.38	0.774
VAP	1.01	1.00, 1.03	0.130	1.08	0.80, 1.46	0.603
BCF	1.05	1.00, 1.10	0.038*	1.18	0.84, 1.65	0.345
ALH	1.10	0.97, 1.25	0.154	0.91	0.12, 6.63	0.925
Normal sperm morphology rate	1.16	1.07, 1.25	<0.001***	0.82	0.48, 1.39	0.463
HAB	0.95	0.92, 0.98	<0.001***	0.57	0.30, 1.06	0.073
MAB	0.98	0.95, 1.00	0.017*	0.55	0.29, 1.01	0.055
PAB	0.97	0.95, 1.00	0.018*	0.55	0.30, 1.02	0.058
CAB	0.97	0.93, 1.02	0.230	0.54	0.29, 1.00	0.051

The multivariate regression analysis hyperactivated spermatozoa revealed significant associations with the adverse pregnancy outcomes, significant protective effect of hyperactivated spermatozoa (adjusted OR, 0.93; 95% CI, 0.87–1.00; *P* = 0.049). No other variables, including age, sperm dynamics parameters, sperm morphology parameters or sperm DNA fragmentation index, reached statistical significance. See **Table 7**.

**Table 7:** Univariate and multivariate analysis of influencing factors (Logistic regression).

Characteristics	Univariable			Multivariable		
	OR	95% CI	*P*	OR	95% CI	*P*
Groups of Obesity						
Non-obesity group	0.87	0.62, 1.23	0.432			
Obesity group	1.19	0.83, 1.70	0.340			
Subgroups of Obesity						
Central obesity group	0.98	0.50, 1.92	0.960			
Simple obesity group	1.21	0.39, 3.79	0.743			
Generalized obesity group	1.19	0.82, 1.72	0.369			
Oligozoospermia	0.93	0.26, 3.34	0.913	1.73	0.45, 6.63	0.423
Asthenospermia	0.68	0.42, 1.08	0.100	1.30	0.65, 2.60	0.461
Teratospermia	0.79	0.56, 1.11	0.170	1.59	0.97, 2.59	0.066
DFI	0.99	0.97, 1.00	0.089	1.00	0.98, 1.01	0.644
Semen volume	1.04	0.96, 1.13	0.364	1.05	0.89, 1.24	0.534
Total sperm count	1.00	1.00, 1.00	0.002**	1.00	1.00, 1.00	0.936
Sperm concentration	1.00	1.00, 1.00	0.002**	1.00	1.00, 1.01	0.393
PR	1.02	1.01, 1.03	<0.001***	1.01	0.96, 1.07	0.723
Hyperactivated spermatozoa	1.03	1.01, 1.05	0.002**	0.93	0.87, 1.00	0.049*
VCL	1.02	1.01, 1.04	<0.001***	0.82	0.65, 1.05	0.122
VSL	1.05	1.03, 1.08	<0.001***	1.31	0.83, 2.05	0.244
VAP	1.04	1.02, 1.06	<0.001***	1.05	0.73, 1.51	0.795
BCF	1.13	1.07, 1.20	<0.001***	1.39	0.93, 2.08	0.103
ALH	1.41	1.20, 1.65	<0.001***	5.20	0.52, 51.79	0.160
Normal sperm morphology rate	1.12	1.03, 1.21	0.008**	0.54	0.28, 1.01	0.052

### 3.5 Analysis of the mediating effect of obesity

Results from the mediation analysis are summarized in **Table 8**. A significant direct effect of obesity on sperm DFI was observed (β = -9.67, 95% CI: -11.19 ~ -8.15, *P* < 0.001), while DFI itself was a significant predictor of adverse pregnancy outcomes (β = -0.02, 95% CI: -0.04 ~ -0.01, *P* = 0.029). The direct path from obesity to adverse pregnancy outcomes was not significant (β = -0.32, 95% CI: -0.69 ~ 0.06, *P* = 0.101), suggesting that the influence of obesity on pregnancy outcomes is primarily mediated through sperm DFI.

**Table 8:** Results of mediation analysis: path analysis.

Path	Relation	*P*	β (95%CI)
Obesity –> DFI	Exposure –> Intermediary	<0.001	-9.67 (-11.19 ~ -8.15)
Obesity –> Adverse pregnancy outcomes	Exposure –> Outcome	0.101	-0.32 (-0.69 ~ 0.06)
DFI –> Adverse pregnancy outcomes	Intermediary –> Outcome	0.029	-0.02 (-0.04~ -0.01)

## 4 Discussion

Obesity has increasingly been recognized as a significant modifier of male reproductive health, with accumulating evidence indicating its adverse effects on conventional semen parameters. Our study reinforces the notion that obesity is associated with diminished sperm quality, particularly manifesting as elevated sperm vitality decreased, abnormality rate and sperm DFI increased. There was no statistically significant difference in total sperm count and sperm concentration between the non-obesity and obesity group populations, while there were differences in sperm dynamics parameters and sperm morphology parameters (*P* < 0.05), which were shown to be poorer in both sperm dynamics parameters in the obesity group population as compared to the non-obesity group population, and morphological parameters. These alterations may be attributed to a multitude of interrelated mechanisms, including chronic systemic inflammation, increased oxidative stress, hormonal imbalances, and scrotal hyperthermia (16–18). The complex interplay of obesity, metabolic syndrome and the reproductive gonadal axis with excess adipose tissue leads to increased conversion of testosterone to oestradiol, resulting in lower testosterone, and lower testosterone - oxidative stress synergism in the testicular microenvironment may lead to reduced sperm counts and sperm DNA damage (19).

Notably, our findings highlight that not all obesity phenotypes exert uniform effects on male fertility outcomes. The stratification of obese individuals into distinct subtypes, generalized obesity, simple obesity and central obesity. There were differences in total sperm count, sperm concentration, sperm dynamics parameters and sperm morphology parameters among obesity subgroups, where central obesity group differed from generalized obesity and simple obesity groups in total sperm count, sperm concentration and normal sperm morphology rate(*P* < 0.05), and showed that sperm dynamics and morphology parameters were better than those of generalized obesity and simple obesity groups. Interestingly and somewhat paradoxically, our results suggest that isolated central obesity might be associated with a protective effect on sperm dynamics and morphological parameters. This finding appears to counter the prevailing consensus that central obesity, in general, is detrimental to male fertility. The relationship between central obesity and male sperm quality seems to have been debated. Many factors such as research population, metabolic diseases, age, etc. For instance, Eisenberg et al. (20) in a study of male population, found no significant associations between male BMI or WC and semen concentration, motility, morphology, or DFI. Wang T et al. (21) concluded that central obesity was significantly associated with a reduction in semen volume, TSC, and total number of motile spermatozoa. Keszthelyi M et al. (22)stated that central obesity has a potential role in progressive viability and TSC, but not in normal morphology and concentration. However, these studies ignored the relationship between central obesity and generalized obesity and failed to separate central obesity from obesity and analyze it in separate subgroups. It is plausible that men who develop isolated abdominal obesity, despite having a normal BMI, represent a distinct metabolic and genetic subgroup. Their ability to maintain a normal overall weight despite central adiposity might be indicative of a more robust metabolic profile, more favorable genetic background in terms of nutrient partitioning, or differences in adipokine secretion patterns compared to men with generalized obesity. Thus, the observed ‘protective’ effect might not be due to the abdominal fat itself, but rather be a marker of this underlying, more favorable constitutive health status that also supports better spermatogenesis. The hormonal milieu in isolated central obesity might be distinct. While generalized obesity is strongly linked to hypogonadism, the impact of isolated visceral fat on testosterone aromatization and estrogen feedback might be different in the absence of overall energy surplus. A specific adipokine or lipid mediator profile from abdominal fat could, theoretically, exert unexpected effects on the testicular microenvironment. WC was significantly increased in Chinese male adults, and age, physical activity, energy intake, alcohol consumption, education, income and were associated with elevated indicators of central obesity (23). We cannot entirely exclude the possibility of residual confounding by unmeasured factors, such as diet quality, physical activity patterns, or genetic polymorphisms that independently influence both body fat distribution and sperm production. This underscores the importance of incorporating anthropometric measures beyond BMI, such as waist circumference, into clinical assessments of male fertility.

No statistically significant differences were observed between the obesity and non-obesity groups regarding the presence of male infertility (57.7% vs. 56.3%, *P* = 0.693) or adverse pregnancy outcomes in partners (22.6% vs. 20.3%, *P* = 0.432). Similarly, the prevalence of oligozoospermia was comparable between groups (1.6% vs. 1.9%, *P* = 0.727). However, significant differences were noted for asthenospermia and teratospermia: the obesity group demonstrated a higher proportion of asthenospermia (26.1% vs. 14.2%, *P* < 0.001) and teratospermia (70.3% vs. 61.4%, *P* = 0.009) compared to the non-obesity group. However, obesity men were more likely to have male infertility (OR = 1.66, 95% CI 1.53-1.79), and the absolute risk of adverse pregnancy outcomes in partners was increased, in addition to increased sperm malformations and DNA fragmentation (24). Obesity is associated with a higher incidence of male factor infertility, including induced sleep apnoea, altered testosterone profiles and increased scrotal temperature (25). In general obese men but not in the population of infertile patients, obesity had no effect on sperm concentration and percentage of normal sperm morphology, but semen volume, total sperm count and sperm viability were reduced (26). There was a statistically significant difference in sperm DFI between obesity and non-obesity groups (*P* < 0.001), and obesity group had higher sperm DFI compared to non-obesity group (23.83 ± 12.25 vs. 14.16 ± 9.80). Increased BMI has been associated with decreased mitochondrial activity in spermatozoa and increased DNA fragmentation (27).However, some studies have also concluded that there is no association between body mass index and sperm DNA integrity (28). There are insufficient data to demonstrate a correlation between BMI and sperm (29).

Results of multivariate regression analysis showed that sperm PR was significantly negatively associated with the risk of male infertility (adjusted OR, 0.93; 95% CI, 0.89-0.98; *P* = 0.004). The multivariate regression analysis hyperactivated spermatozoa revealed significant associations with the adverse pregnancy outcomes, significant protective effect of hyperactivated spermatozoa (adjusted OR, 0.93; 95% CI, 0.87~1.00; *P* = 0.049). It was demonstrated that sperm motility (PR and hyperactivated spermatozoa) decreases the probability of male infertility and adverse pregnancy outcomes, thus flanking the fact that sperm motility indicators tend to predict the overall quality of spermatozoa, which can lead to good pregnancy outcomes. A significant direct effect of obesity on sperm DFI was observed (β = -9.67, 95% CI: -11.19 ~ -8.15, *P* < 0.001), while DFI itself was a significant predictor of adverse pregnancy outcomes (β = -0.02, 95% CI: -0.04 ~ -0.01, *P* = 0.029). The direct path from obesity to adverse pregnancy outcomes was not significant (β = -0.32, 95% CI: -0.69 ~ 0.06, *P* = 0.101), suggesting that the influence of obesity on pregnancy outcomes is primarily mediated through sperm DFI. Obesity leads to an increased risk of sperm DNA damage (30). In turn, sperm DNA damage is associated with reduced fertilization rates, embryo quality and pregnancy rates, as well as an increased incidence of spontaneous abortion (31). In addition to signs and symptoms directly stemming from decreased circulating testosterone levels, obese men have poor fertility, further demonstrating the parallel between obesity and male reproductive function (32). In addition, androgen-deficient men exhibit increased fat accumulation, resulting in a vicious cycle of obesity-decreased androgen-fat accumulation. Male hypogonadism is usually associated with testosterone deficiency, impaired spermatogenesis, and metabolic disorders such as obesity (33). Metabolic disorders in childhood, late pubertal metabolic syndrome, and late pubertal development have been associated with impaired testicular function in adulthood, such as decreased sperm count and quality and decreased testosterone levels (34–36). While sex hormones and testicular factors are critical, obesity may have a direct impact on sperm quality through mechanisms such as insulin resistance, chronic inflammation or oxidative stress. Exercising at moderate intensity and eating a healthy diet can improve semen quality and fertility and have a positive impact on male reproductive health (37, 38).

The aim of this study was to investigate the effects of obesity and different obesity types, especially body fat distribution site characteristics, on routine semen dynamics and morphological parameters and sperm DNA integrity in men. Male infertility is a global health problem and, at the same time, the prevalence of obesity is increasing dramatically. Although a large number of cross-sectional studies have clarified the association between obesity and reduced semen quality, most of them have relied solely on BMI for diagnosis, ignoring the inherent heterogeneity of obesity. BMI, as a total body weight index, is unable to differentiate between adiposity and muscularity, let alone reflecting the distribution of body fat, a factor which has proved to be of paramount importance in metabolic disorders. The present study innovatively subdivided the obesity into generalized obesity, simple obesity and central obesity, and the results suggest that central obesity may better sperm morphology and viability, a breakthrough from traditional perception. This result suggests that the accumulation of abdominal fat, rather than simply being overweight in terms of total body weight, may not be the key factor driving impaired sperm quality. The underlying mechanisms of central obesity alone may be related to metabolic factors such as positive nitrogen balance nutritional status and sleep quality. This finding not only deepens our understanding of obesity-mediated fertility, but also highlights the need to incorporate waist circumference into clinical assessment, providing new perspectives on fertility assessment and personalized interventions for men with specific obesity phenotypes. The present study revealed significant heterogeneity in the association between obesity and semen quality parameters in men through meticulous body fat distribution typing.

### 4.1 Summary

The present study summarizes that obesity reduces sperm quality (sperm dynamics and sperm morphology parameters) and may lead to male infertility and adverse pregnancy outcomes. Central obesity can be used as an assessment of sperm quality and fertility in men, but in combination with many confounding factors. The results of the present study include obesity among the modifiable risk factors for male fertility, and the obesity classification provides a complete and systematic understanding of male fertility and sperm function.

### 4.2 Study innovations

The main innovation of this study is the refinement and deepening of the analysis of obesity and male semen parameters. Unlike most of the previous studies, which only classified obesity as binary (obese/non-obese) based on body mass index, the present study adopted a more precise multidimensional obesity phenotype stratification strategy. This study not only compared the overall differences between the obese and non-obese groups, but also further distinguished the obese group into three subtypes with different metabolic and somatic characteristics: generalized obesity, simple obesity, and central obesity. This classification effectively reveals the different pathophysiological mechanisms that may exist within heterogeneous obesity and avoids the bias associated with considering obesity as a homogeneous group. On this basis, we innovatively explored the specific associations between these specific obese subtypes and key semen quality parameters such as sperm morphology parameters, sperm dynamics parameters and sperm DNA fragmentation index. This provides a more precise insight into the specific ways in which obesity affects male fertility, suggesting that different types of body fat distributions may have differential effects on spermatogenesis through different mechanisms (e.g., hormonal disturbances, oxidative stress). The results of this study are expected to provide an important theoretical basis for future personalized fertility interventions for specific obese populations.

### 4.3 Study limitations

Several shortcomings remain in this study. Firstly, the cross-sectional study design was unable to determine a causal relationship between obesity and semen parameters, and attempted to control for a variety of known metabolic factors, but could not completely exclude or potential or undetected confounders, and residual confounders may have affected the results of the study, which need to be further validated in the future through prospective cohort studies or intervention trials. Our analysis relied on conventional anthropometric indices like BMI and WC. While informative, BMI does not distinguish between lean mass and adipose tissue, and neither BMI nor WC fully captures the complex three-dimensional distribution of body fat. Novel indices, such as the A Body Shape Index (ABSI) and Body Roundness Index (BRI), have been developed to better reflect body shape and regional adiposity (39). Future studies would benefit from incorporating these novel indices to provide a more nuanced understanding of the relationship between body composition and sperm parameters. In addition, the study did not systematically collect factors such as sperm retrieval season, number of days of abstinence, dietary structure, frequency of exercise, and quality of sleep of the participants, which may affect sperm quality as additional confounding variables. Finally, all samples came from the same center, which may have limitations in sample size and population representativeness, and future multi-center and large-sample studies are needed to enhance the generalizability of the findings.

## Data Availability

The original contributions presented in the study are included in the article/**Supplementary Material**. Further inquiries can be directed to the corresponding author/s.

## Supplementary material

Supplementary information 1 The **Supplementary Material** were mainly the sex hormones (T, E2, FSH,PRL, LH) and testicular volume of some subjects. Because if the semen routine analysis is normal in the early stage, there is often no further sex hormone examination (clinical diagnosis and treatment logic), and study limitations, so only a small number of research objects have sex hormone examination. In order to increase the authenticity of the research conclusion, this part of the baseline data is added to the **Supplementary Material**.

## References

[1] GBD 2021 Adult BMI Collaborators. Global, regional, and national prevalence of adult overweight and obesity, 1990-2021, with forecasts to 2050: a forecasting study for the Global Burden of Disease Study 2021. Lancet. (2025) 405:813–38. doi: 10.1016/S0140-6736(25)00355-1, PMID: 40049186 PMC11920007

[2] Wang L Zhou B Zhao Z Yang L Zhang M Jiang Y. Body-mass index and obesity in urban and rural China: findings from consecutive nationally representative surveys during 2004-18. Lancet. (2021) 398:53–63. doi: 10.1016/S0140-6736(21)00798-4, PMID: 34217401 PMC7617101

[3] Shen C Zhou Z Lai S Tao X Zhao D Dong W. Urban-rural-specific trend in prevalence of general and central obesity, and association with hypertension in Chinese adults, aged 18-65 years. BMC Public Health. (2019) 19:661. doi: 10.1186/s12889-019-7018-4, PMID: 31146734 PMC6543650

[4] Du T Sun X Yin P Huo R Ni C Yu X. Increasing trends in central obesity among Chinese adults with normal body mass index, 1993-2009. BMC Public Health. (2013) 13:327. doi: 10.1186/1471-2458-13-327, PMID: 23575244 PMC3626835

[5] Sun X Liu Z Du T. Secular trends in the prevalence of abdominal obesity among Chinese adults with normal weight, 1993-2015. Sci Rep. (2021) 11:16404. doi: 10.1038/s41598-021-95777-y, PMID: 34385525 PMC8360975

[6] Vander Borght M Wyns C. Fertility and infertility: Definition and epidemiology. Clin Biochem. (2018) 62:2–10. doi: 10.1016/j.clinbiochem.2018.03.012, PMID: 29555319

[7] Levine H Jørgensen N Martino-Andrade A Mendiola J Weksler-Derri D Jolles M. Temporal trends in sperm count: a systematic review and meta-regression analysis of samples collected globally in the 20th and 21st centuries. Hum Reprod Update. (2023) 29:157–76. doi: 10.1093/humupd/dmac035, PMID: 36377604

[8] Cipriani S Ricci E Chiaffarino F Esposito G Dalmartello M Vecchia CL. Trend of change of sperm count and concentration over the last two decades: A systematic review and meta-regression analysis. Andrology. (2023) 11:997–1008. doi: 10.1111/andr.13396, PMID: 36709405

[9] Lv MQ Ge P Zhang J Yang YQ Zhou L Zhou DX. Temporal trends in semen concentration and count among 327–373 Chinese healthy men from 1981 to 2019: a systematic review. Hum Reprod. (2021) 36:1751–75. doi: 10.1093/humrep/deab124, PMID: 34046659

[10] Mann U Shiff B Patel P. Reasons for worldwide decline in male fertility. Curr Opin Urol. (2020) 30:296–301. doi: 10.1097/MOU.0000000000000745, PMID: 32168194

[11] Santi D Lotti F Sparano C Rastrelli G Isidori AM Pivonello R. Does an increase in adipose tissue ‘weight’ affect male fertility? A systematic review and meta-analysis based on semen analysis performed using the WHO 2010 criteria. Andrology. (2024) 12:123–36. doi: 10.1111/andr.13460, PMID: 37226894

[12] Anifandis G Dafopoulos K Messini CI Polyzos N Messinis IE. The BMI of men and not sperm parameters impact on embryo quality and the IVF outcome. Andrology. (2012) 1:85–9. doi: 10.1111/j.2047-2927.2012.00012.x, PMID: 23258634

[13] Nikolic AZ Dragojevic-Dikic S Kocic J Babic U Joksimovic A Radakovic-Cosic J. Influence of male body mass index on semen analysis parameters and *in vitro* fertilization outcomes. Med (Baltimore). (2024) 103:e38949. doi: 10.1097/MD.0000000000038949, PMID: 39093753 PMC11296464

[14] Guo D Wu W Tang Q Qiao S Chen Y Chen M. The impact of BMI on sperm parameters and the metabolite changes of seminal plasma concomitantly. Oncotarget. (2017) 8:48619–34. doi: 10.18632/oncotarget.14950, PMID: 28159940 PMC5564712

[15] Service CA Puri D Al Azzawi S Hsieh TC Patel DP. The impact of obesity and metabolic health on male fertility: a systematic review. Fertil Steril. (2023) 120:1098–111. doi: 10.1016/j.fertnstert.2023.10.017, PMID: 37839720

[16] Pini T Parks J Russ J Dzieciatkowska M Hansen KC Schoolcraft WB. Obesity significantly alters the human sperm proteome, with potential implications for fertility. J Assist Reprod Genet. (2020) 37:777–87. doi: 10.1007/s10815-020-01707-8, PMID: 32026202 PMC7183029

[17] Fan W Xu Y Liu Y Zhang Z Lu L Ding Z. Obesity or overweight, a chronic inflammatory status in male reproductive system, leads to mice and human subfertility. Front Physiol. (2018) 8:1117. doi: 10.3389/fphys.2017.01117, PMID: 29354072 PMC5758580

[18] Garolla A Torino M Miola P Caretta N Pizzol D Menegazzo M. Twenty-four-hour monitoring of scrotal temperature in obese men and men with a varicocele as a mirror of spermatogenic function. Hum Reprod. (2015) 30:1006–13. doi: 10.1093/humrep/dev057, PMID: 25779699

[19] Michalakis K Mintziori G Kaprara A Tarlatzis BC Goulis DG. The complex interaction between obesity, metabolic syndrome and reproductive axis: a narrative review. Metabolism. (2013) 62:457–78. doi: 10.1016/j.metabol.2012.08.012, PMID: 22999785

[20] Eisenberg ML Kim S Chen Z Sundaram R Schisterman EF Buck Louis GM. The relationship between male BMI and waist circumference on semen quality: data from the LIFE study. Hum Reprod. (2014) 29:193–200. doi: 10.1093/humrep/det428, PMID: 24306102 PMC3896223

[21] Wang T Wang Q Fan Z Xu R Deng X Li Y. Association between central obesity and semen quality: A cross-sectional study in 4513 Chinese sperm donation volunteers. Andrology. (2024) 12:316–26. doi: 10.1111/andr.13471, PMID: 37282772

[22] Keszthelyi M Gyarmathy VA Kaposi A Kopa Z. The potential role of central obesity in male infertility: body mass index versus waist to hip ratio as they relate to selected semen parameters. BMC Public Health. (2020) 20:307. doi: 10.1186/s12889-020-8413-6, PMID: 32164645 PMC7066798

[23] Qian X Su C Zhang B Qin G Wang H Wu Z. Changes in distributions of waist circumference, waist-to-hip ratio and waist-to-height ratio over an 18-year period among Chinese adults: a longitudinal study using quantile regression. BMC Public Health. (2019) 19:700. doi: 10.1186/s12889-019-6927-6, PMID: 31170949 PMC6555739

[24] Campbell JM Lane M Owens JA Bakos HW. Paternal obesity negatively affects male fertility and assisted reproduction outcomes: a systematic review and meta-analysis. Reprod BioMed Online. (2015) 31:593–604. doi: 10.1016/j.rbmo.2015.07.012, PMID: 26380863

[25] Du Plessis SS Cabler S McAlister DA Sabanegh E Agarwal A. The effect of obesity on sperm disorders and male infertility. Nat Rev Urol. (2010) 7:153–61. doi: 10.1038/nrurol.2010.6, PMID: 20157305

[26] Wang S Sun J Wang J Ping Z Liu L. Does obesity based on body mass index affect semen quality?-A meta-analysis and systematic review from the general population rather than the infertile population. Andrologia. (2021) 53:e14099. doi: 10.1111/and.14099, PMID: 34028074

[27] Fariello RM Pariz JR Spaine DM Cedenho AP Bertolla RP Fraietta R. Association between obesity and alteration of sperm DNA integrity and mitochondrial activity. BJU Int. (2012) 110:863–7. doi: 10.1111/j.1464-410X.2011.10813.x, PMID: 22300410

[28] Bandel I Bungum M Richtoff J Malm J Axelsson J Pedersen HS. No association between body mass index and sperm DNA integrity. Hum Reprod. (2015) 30:1704–13. doi: 10.1093/humrep/dev111, PMID: 25994665

[29] Sepidarkish M Maleki-Hajiagha A Maroufizadeh S Rezaeinejad M Almasi-Hashiani A Razavi M. The effect of body mass index on sperm DNA fragmentation: a systematic review and meta-analysis. Int J Obes (Lond). (2020) 44:549–58. doi: 10.1038/s41366-020-0524-8, PMID: 31949297

[30] Dupont C Faure C Sermondade N Boubaya M Eustache F Clément P. Obesity leads to higher risk of sperm DNA damage in infertile patients. Asian J Androl. (2013) 15:622–5. doi: 10.1038/aja.2013.65, PMID: 23792341 PMC3881654

[31] Lewis SE John Aitken R Conner SJ Iuliis GD Evenson DP Henkel R. The impact of sperm DNA damage in assisted conception and beyond: recent advances in diagnosis and treatment. Reprod BioMed Online. (2013) 27:325–37. doi: 10.1016/j.rbmo.2013.06.014, PMID: 23948450

[32] Carrageta DF Oliveira PF Alves MG Monteiro MP. Obesity and male hypogonadism: Tales of a vicious cycle. Obes Rev. (2019) 20:1148–58. doi: 10.1111/obr.12863, PMID: 31035310

[33] Pivonello R Menafra D Riccio E Garifalos F Mazzella M de Angelis C. Metabolic disorders and male hypogonadotropic hypogonadism. Front Endocrinol (Lausanne). (2019) 10:345. doi: 10.3389/fendo.2019.00345, PMID: 31402895 PMC6669361

[34] Wagner IV Oliver E Dötsch J Söder O. Adverse effects of metabolic disorders in childhood on adult reproductive function and fertility in the male. J Pediatr Endocrinol Metab. (2020) 34:13–23. doi: 10.1515/jpem-2020-0276, PMID: 33185575

[35] Hart RJ Doherty DA Mori TA Adams LA Huang RC Minaee N. Features of the metabolic syndrome in late adolescence are associated with impaired testicular function at 20 years of age. Hum Reprod. (2019) 34:389–402. doi: 10.1093/humrep/dey371, PMID: 30576537

[36] Jensen TK Finne KF Skakkebæk NE Andersson AM Olesen IA Joensen UN. Self-reported onset of puberty and subsequent semen quality and reproductive hormones in healthy young men. Hum Reprod. (2016) 31:1886–94. doi: 10.1093/humrep/dew122, PMID: 27270973

[37] Zańko A Pawłowski M Milewski R. The impact of physical exercise on male fertility through its association with various processes and aspects of human biology. J Clin Med. (2025) 14:3442. doi: 10.3390/jcm14103442, PMID: 40429435 PMC12112722

[38] Salas-Huetos A Bulló M Salas-Salvadó J. Dietary patterns, foods and nutrients in male fertility parameters and fecundability: a systematic review of observational studies. Hum Reprod Update. (2017) 23:371–89. doi: 10.1093/humupd/dmx006, PMID: 28333357

[39] Nikolic A Mikovic Z Gerginic V Dragojevic DS Bojovic JD Salovic B. Correlation of A body shape index and body roundness index as novel anthropometric indices with semen analysis parameters and IVF outcomes. Am J Mens Health. (2025) 19:15579883251380206. doi: 10.1177/15579883251380206, PMID: 41025204 PMC12484924

