# Supplementary material for: Beyond body mass index: the role of fat distribution in male sperm quality
Source: Front Endocrinol (Lausanne). 2025 Oct 23;16:1702791. doi: 10.3389/fendo.2025.1702791 (PMC12559801; doi:10.3389/fendo.2025.1702791)
Supplement: Supplementary information 1 — The Supplementary Material were mainly the sex hormones (T, E2, FSH,PRL, LH) and testicular volume of some subjects. Because if the semen routine analysis is normal in the early stage, there is often no further sex hormone examination (clinical diagnosis and treatment logic), and study limitations, so only a small number of research objects have sex hormone examination. In order to increase the authenticity of the research conclusion, this part of the baseline data is added to the Supplementary Material . [file DataSheet1.pdf]

Supplementary information 1

Comparison of Sex Hormone Profiles and Testicular Volume Across Obesity Phenotypes

Comparative analysis of sex hormone profiles and testicular volume revealed no statistically significant differences between the obesity and non-obesity groups, nor among the obesity subgroups based on fat distribution patterns (Table S1).

No significant differences were observed between the obesity and non-obesity groups in testosterone levels (15.53 ± 9.72 vs. 13.92 ± 6.30 nmol/L, $P = 0.281$), estradiol (112.58 ± 39.70 vs. 115.83 ± 39.85 pmol/L, $P = 0.613$), follicle-stimulating hormone (median [IQR]: 4.29 [3.22, 6.69] vs. 4.10 [2.81, 6.47] IU/L, $P = 0.416$), prolactin (10.24 [7.95, 14.93] vs. 10.43 [7.66, 13.71] μg/L, $P = 0.720$), luteinizing hormone (3.71 [2.68, 4.82] vs. 3.69 [2.80, 4.82] IU/L, $P= 0.805$), or testicular volume (14.32 ± 1.78 vs. 14.33 ± 1.94 ml, $P = 0.981$).

Subgroup analysis comparing generalized obesity, simple obesity, and central obesity patterns similarly demonstrated no statistically significant differences in any of the measured sex hormone parameters or testicular volume (All $P > 0.05$).

Table S1 Comparison of sex hormone and testicular volume between obesity groups and subgroups

Grouping	T	E2	FSH	PRL	LH	Testicular volume
Groups						
Obesity group	15.53±9.72	112.58±39.70	4.29(3.22,6.69)	10.24(7.95,14.93)	3.71(2.68,4.82)	14.32±1.78
Non-obesity group	13.92±6.30	115.83±39.85	4.10(2.81,6.47)	10.43(7.66,13.71)	3.69(2.80,4.82)	14.33±1.94
t/Z	1.089	0.506	-0.813	-0.383	-0.247	0.024
P	0.281	0.613	0.416	0.720	0.805	0.981
Subgroups						
Generalized obesity group	13.83±6.25	112.12±38.39	3.87(2.79,5.70)	10.76(8.00,14.09)	3.68(2.72,4.81)	14.27±2.24
Simple obesity group	14.47±6.12	113.93±49.97	5.10(3.55,8.33)	9.45(6.99,13.25)	3.72(2.84,4.92)	14.17±1.62
Central obesity group	13.86±6.83	113.60±37.94	4.26(3.14,7.81)	9.34(7.49,12.55)	3.63(3.00,4.80)	14.58±1.68
F/H	0.110	0.035	3.580	2.025	0.247	0.655
P	0.896	0.996	0.167	0.363	0.808	0.721

T:Testosterone(nmol/L), E2: Estradiol(pmol/L), FSH:Follicle-Stimulating Hormone(IU/L), PRL:Prolactin(ug/L),LH:Luteinizing Hormone(IU/L), Testicular volume (ml)
